# Prediction of Human Intestinal Absorption by GA Feature Selection and Support Vector Machine Regression

**DOI:** 10.3390/ijms9101961

**Published:** 2008-10-20

**Authors:** Aixia Yan, Zhi Wang, Zongyuan Cai

**Affiliations:** State Key Laboratory of Chemical Resource Engineering, Department of Pharmaceutical Engineering, P.O. Box 53, Beijing University of Chemical Technology, 15 BeiSanHuan East Road, Beijing 100029, P.R. China. E-Mails: zenowangzhi@126.com (Z. W.); caizongyuancn@yahoo.com (Z. C.)

**Keywords:** Human intestinal absorption (HIA), Kohonen’s self-organizing Neural Network (KohNN), Support Vector Machine (SVM), Genetic Algorithm Feature Selection, Quantitative Structure Activity Relationships (QSAR)

## Abstract

QSAR (Quantitative Structure Activity Relationships) models for the prediction of human intestinal absorption (HIA) were built with molecular descriptors calculated by ADRIANA.Code, Cerius^2^ and a combination of them. A dataset of 552 compounds covering a wide range of current drugs with experimental HIA values was investigated. A Genetic Algorithm feature selection method was applied to select proper descriptors. A Kohonen's self-organizing Neural Network (KohNN) map was used to split the whole dataset into a training set including 380 compounds and a test set consisting of 172 compounds. First, the six selected descriptors from ADRIANA.Code and the six selected descriptors from Cerius^2^ were used as the input descriptors for building quantitative models using Partial Least Square (PLS) analysis and Support Vector Machine (SVM) Regression. Then, another two models were built based on nine descriptors selected by a combination of ADRIANA.Code and Cerius^2^ descriptors using PLS and SVM, respectively. For the three SVM models, correlation coefficients (r) of 0.87, 0.89 and 0.88 were achieved; and standard deviations (s) of 10.98, 9.72 and 9.14 were obtained for the test set.

## 1. Introduction

In drug discovery and development process, complexity and risk have increased greatly as they have become more expensive and time-consuming. Hundreds of millions of dollars and several years are required to develop a new drug. Once on the market, some drugs fail to recover their research and development costs. Market withdrawals add to the industry’s problems. The attrition of compounds through clinical development means that only one in ten compounds entering development will ever make it to the marketplace [1].

The main cause for high attrition rates in drug discovery is from the absorption, distribution, metabolism and excretion (ADME) properties of candidate compounds. Many active drugs fail in phase II or III of the clinical development process because they do not reach their intended target. Poor ADME properties are the major reason for failures. So absorption, distribution, metabolism and elimination studies have to be carefully considered in the drug discovery process, and better ADME properties are pursued by getting experimental data through high throughput screening.

Human intestinal absorption (HIA) is one of the most important ADME properties. Utilization of drugs in the human body is such a complicated process that it can hardly be analyzed precisely by statistical models. HIA is also one of the key steps during the drugs’ transporting to their targets. In addition, it is difficult to predict oral bioavailability for diverse sets of pharmaceuticals, because there are various components playing a role in this process [2]. Due to the diverse pathways of absorption of drugs, powerful descriptors related to carrier-mediated transport and first-pass metabolism are needed for building a useful prediction model for human oral bioavailability. And HIA is considered as one of the important components which influence bioavailability, so a lot of effort has been made for accurate prediction of HIA.

Drug molecules are transported from the gastroenteric tract to the blood circle and permeate the gastroenteric membrane by various mechanisms. The primary mechanism is passive diffusion caused by a concentration gradient. P-Glycoprotein (P-gp) is a common carrier in drugs intestinal penetration, which caused efflux process. This process has been discussed in previous articles: Varma and colleagues evaluated the quantitative contribution of passive permeability to P-glycoprotein-mediated (P-gp-mediated) efflux [3]. The functional activity of P-gp in determining intestinal absorption of drugs was also evaluated. A Biopharmaceutics Classification System was used to classify 63 P-gp substrates (P-gpS) and 73 nonsubstrates (NS) into three classes. Xue and colleagues used support vector machines (SVM) with recursive feature elimination (RFE) to build P-gp classification model [4].

Some researchers have made predictions of human intestinal absorption from molecular graph- based models. A typical application was made by Klopman and colleagues [5]; they built a HIA model with 37 structural descriptors derived from the chemical structures for a data set of 417 drugs. The model was able to predict the percentage of drug absorbed from the gastrointestinal tract. Pérez and colleagues used a topological sub-structural approach (TOPS-MODE) to classify HIA properties into three classes (<30%, 30%–79%, >80%) [6]. Two linear discriminate analyses were carried out. An external prediction set of 127 drugs and a test set of 109 oral drugs with bioavailability values were reported. Sun and colleagues predicted LogP, LogS, LogBB, and HIA by atom type classification and partial least-squares (PLS) method [7]. The five-component PLS-DA HIA model separated the compounds into three classes.

The most frequently used approaches to make QSAR (quantitative structure activity relationship) predictions involve artificial methods such as evolution algorithms or artificial neural networks (ANN). Many applications have been proposed in previous papers. Wessel and colleagues developed a QSAR model for the prediction HIA values by a genetic algorithm combined with a neural network fitness evaluator based on 86 drugs and drug-like compounds [8]. The molecules were encoded with calculated molecular structure descriptors including charge and bond descriptors. Zhao and colleagues built models on 169 compounds [9, 10]. Five descriptors called ‘Abraham descriptors’ were derived from their ABSOLV program, which correspond to basic physicochemical properties. A reliable model was constructed for 38 compounds; another model for the total 169 compounds was also built. Cruciani and colleagues modeled the BBB and caco-2 cell absorption properties of 35 compounds with VolSurf descriptors which refer to molecular size and shape, to size and shape of both hydrophilic and hydrophobic regions and to the balance between them [11]. Kustrin and colleagues applied genetic neural network (GNN) to model HIA properties of 83 drugs [12]. The 15 descriptors involved polarity, hydrogen bonding, and conformational stabilities. Osterberg and colleagues applied PLS statistics to predict biopharmaceutical properties including HIA from ACD/ChemSketch and ACD/logP descriptors [13]. Norinder used MolSurf descriptors and multivariate partial least squares projections to latent structures [14]. Niwa and colleagues built a HIA model of 86 compounds based on their 2D descriptors [15]. A general regression neural network (GRNN) and a probabilistic neural network (PNN) were applied. Wegner and colleagues used an adaptive boosting algorithm to solve the binary classification problem (AdaBoost.M1) and Genetic Algorithms based on Shannon Entropy Cliques (GA-SEC) variants as hybrid feature selection algorithms [16]. The model was got from 52 drugs and TPSA (JOELib) descriptors.

This work aimed at building reliable QSAR models for predicting compound HIA using physico-chemical descriptors calculated from a compound’s structure. The procedure includes: (1) a structure dataset is set up with experimental HIA values; (2) descriptors are calculated by the descriptor generators ADRIANA.Code 2.1 [17–18] and Cerius^2^ 4.10L [19]; (3) subsets of descriptors are selected by the Genetic Algorithms program genetic-PLS [20]; (4) the dataset is divided into training set and test set by Kohonen's self-organizing neural network [21]; (5) using the Partial Least Square (PLS) method and the Support Vector Machine (SVM) program Libsvm [22] models are built with training the set and tested with the test set.

## 2. Data Sets

The data for human intestinal absorption were derived from Hou’s dataset (Training_set_454.sdf and Test_set_98.sdf) [23]. Altogether 552 compounds were available for passive diffusion analysis. Abraham had provided us with a dataset of 241 compounds with HIA values and SMILES structures [9] before Hou’s data were obtained; other HIA data from the literature were also collected [5, 7, 11, 14, 15]. After our examination, the data from Abraham and other literature were contained in Hou’s dataset, so Hou’s dataset was adopted in our study. All chemical structures of the compounds in the dataset (especially the chirality) were checked against the following databases: National Library of Medicine [24], ChemFinder database [25], and Chemblink database [26].

For the ultimate dataset with 552 compounds, molecular weight (MW) was distributed in the range of 46 to 1403, octanol-water partitioning coefficient (Log P) was distributed in the range of −17.83 to 9.71, and HIA (%) value was distributed in the range of 0 to 100.

## 3. Methods

### 3.1. Descriptors

A total number of 107 descriptors were calculated. They were calculated by the ADRIANA. Code 2.1 [17, 18] and Cerius^2^ 4.10L [19].

Fifty five descriptors were calculated by ADRIANA.Code. they include: molecular weight (MW), Topological Polar Surface Area (TPSA) [27], aqueous solubility (log*S*) [17, 28, 29], octanol/water partition coefficient (Xlog*P*) [30], number of violations of the rule of 5 (N_rule5_) [31], number of H-bond donor groups (H_don_), number of H-bond acceptor groups (H_acc_), 2D molecular autocorrelation vectors *et al*.

In the autocorrelation vectors calculated by ADRIANA. Code, the hydrogen atoms were included. 2D molecular autocorrelation vectors [32] for physicochemical atomic properties were calculated for each molecule by using the following equation:

(1) $$A(d)=\sum_{ij}p_{i}p_{j}({\text{d}=\text{d}}_{\text{j}}{-\text{d}}_{\text{i}})$$

where *A(d*) is the topological autocorrelation coefficient referring to atom pairs *i*, *j* which are separated by *d* bonds. *pi* is an atomic property, e.g. the σ charge on atom *i*. Thus, for each compound, a series of coefficients for different topological distances *d*, a so-called autocorrelation vector is obtained; Seven distances from distance of *d*=0 to *d*=6 were considered. Seven atomic properties are represented by *pi:*·σ charge;(SigChg) [33–34], π charge (PiChg) [35], total charges (TotChg), σ electronegavity (SigEN), π elctronegativity (PiEN), lone-pair electronegativity (LpEN) and atomic polarizability (Apolariz) [36].

**Figure 1.**
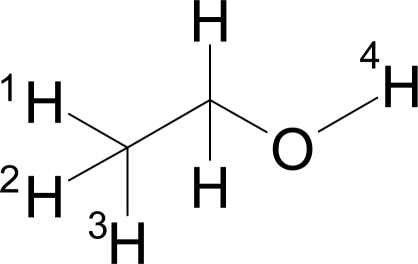
An example for autocorrelation coefficient calculation.

For example, ethanol (Figure 1) has three pairs of atoms that are separated by four bonds: H_1_–H_4_, H_2_–H_4_ and H_3_–H_4_. Thus, the corresponding autocorrelation for the topological distance four computes to:

(2) $$A(4)=p_{1}p_{4}+p_{2}p_{4}+p_{3}p_{4}$$

The other 52 descriptors were calculated by Cerius2 4.10L as follows: molecular weight (MW), number of rotatable bonds (N_rot_), number of H-bond donor groups (H_don_), number of H-bond acceptor groups (H_acc_), octanol-water partitioning coefficient (LogP), molecular molar volume, molecular molar refractivity (MR), number of violations of the rule of 5 (N_rule5_) [31], radius of gyration, molecular area, molecular volume, principal moment of inertia, 10 shadow indices, 12 Kier and Hall molecular connectivity indices (ø), Wiener index (W), and Zagreb index (Zagreb) *et al*. [37].

It is commonly considered that TPSA, LogP, N_rot_, Nrule5 are responsible descriptors for HIA prediction. In order to evaluate the performance of descriptors calculated by ADRIANA.Code and descriptors calculated by Cerius^2^, two sets of descriptors were taken into models separately. The mixture of two sets of descriptors was then also used for building models that may have good quality for predicting HIA. Thus the dataset with 552 compounds was converted into three datasets with different descriptors.

### 3.2. Feature Selection of the Descriptors with GA Strategy

A program genetic-PLS^20^ was applied to select the proper descriptors in this work. This tool can be run in a MatLab environment (MatLab version 4.0 and later versions). This is an optimization software based on the GA strategy and the its principles can be described as follows: (1) definition and encoding; (2) reaction of initial population; (3) evaluation of each chromosome; (4) protection of chromosome; (5) selection of best chromosomes; (6) crossover and mutation; (7) stop if a halt condition is satisfied, otherwise go to step 3. Three functions included in this program were employed in our study: GAPLSOPT(1), GAPLSOPT(2), GAPLS. GAPLSOPT(1) was used for testing whether the dataset was suitable to this study. GAPLSOPT(2) was used to estimate the number of evaluations that was required in the function GAPLS. GAPLS was run in order to select descriptors. More details about the principles of this GA strategy can be found in Leardi’s articles [38–40]. The author had studied feature optimization of spectral data with his genetic-PLS tools. The results proved this tool could accomplish the feature selection job successfully [41].

Three sets of descriptors (descriptors calculated by Cerius^2^, descriptors calculated by ADRIANA. Code and the combination of them) were adopted in genetic-PLS selection respectively. Therefore three corresponding sets of selected descriptors were obtained. In order to decrease interferes of multicollinearity before genetic-PLS selection, for each pair of descriptors with correlation coefficients over 0.9 in one set of descriptors, only one descriptor remained. The detailed procedure of genetic-PLS was demonstrated with descriptors calculated by Cerius^2^. The other two selection procedures were very similarly. The parameters were set to defaults [41]. Here the 52 Cerius^2^ descriptors were taken into the genetic-PLS selection, as an example.

**Figure 2.**
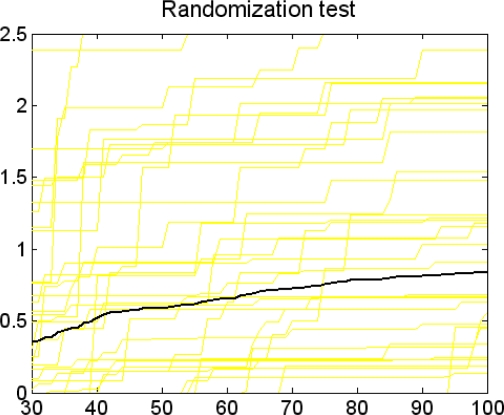
GAPLSOPT(1) test

Before the feature selection could be started, some preparations were needed by using the functions GAPLSOPT(1) and GAPLSOPT(2). GAPLSOPT(1) could be used for testing whether the dataset was suitable to this program. According to the author’ presentation: good datasets got results < 5; datasets with results < 10 was acceptable for GA robusty. When 52 Cerius^2^ descriptors and corresponding HIA data were tested by GAPLSOPT(1), as Figure 2 shows, the result of GAPLSOPT(1) test (dark line) spanned from 0.3 to 1.So the dataset was suitable to this program [41].

**Figure 3.**
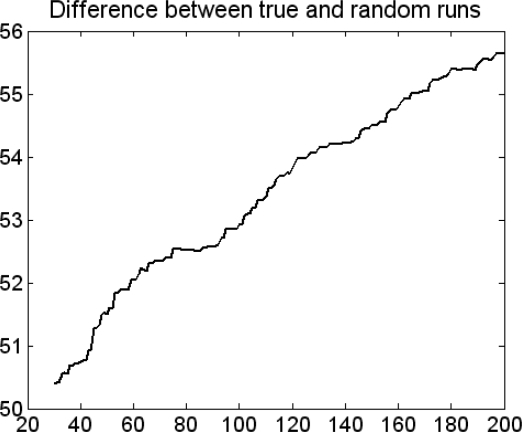
GAPLSOPT(2)differences curve.

GAPLSOPT(2) was used to estimate the number of evaluations that was required in the function GAPLS. The way to find the best number of evaluations was to pick the point where no significant increase is observed. Normally the value should be controlled between 50 and 200 to prevent overfitting. As Figure 3 shows, the GAPLSOPT(2) differences curve is a continuous ascending curve. The number of evaluations was thus set to its maximum value 200.

**Figure 4.**
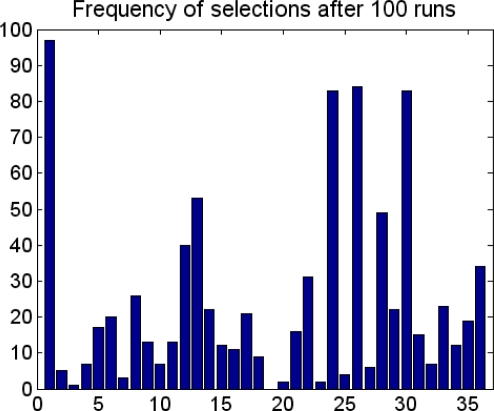
Select frequency figure by GAPLS function. Five repetitions were executed to obtain an average result.

After these preparations, GAPLS could be run to make feature selection. To reduce the random errors, five repeats of GAPLS were applied; each includes 100 runs, which was set by the program. Figure 4 showed the cross validation response and the select frequency of all 52 Cerius^2^ descriptors. The six most frequently used descriptors were chosen for further analysis.

### 3.3. Training/Test Set Selection with Kohonen's Self-organizing Neural Network

Kohonen’s self-organizing Neural Network (KohNN) [21] has the special property of effectively creating a spatially organized internal representation of various features of input signals and their abstractions. A two-dimensional array map with neurons was then generated to classify the dataset. Data with similar input were mapped into the same neuron or neighbor neurons in the neural network.

The dataset was split into training set and test set with the generated feature map. This division had an advantage compared to random selection [42–43]. This method is for splitting a data set into training set and test set, and assures that both sets cover the information space as good as possible. As the test set was not used during training of the PLS or SVM model, it still can be considered as an external dataset.

### 3.4. Support Vector Machine (SVM) Analysis

The Libsvm program was used to build SVM models [22]. This software is based on the function of classification. After some improvement, it can also be applied to the regression problem well. More introductions and implementations about Libsvm can be found in their website [44–45]. The Libsvm regression was realized by the ɛ-Support Vector Regression (ɛ-SVR) with a radial basis function (RBF) kernel function. The ɛ-SVR algorithm is a generalization of the better known support vector classification algorithm to the regression case. Given *n* training vectors *x_i_* and a vector *y* ∈*Rn* such that *y* ∈*R* , we want to find an estimate for the function *y* = f(*x*) which is optimal from a structural risk minimization viewpoint. According to ɛ-SVR, this estimate is:

(3) $$f(x)=\sum_{i=1}^{n}\left(a_{i}^{*}-a_{i}\right)k(x_{i},x_{j})+b$$

where *b* is a bias term and k(*x_i_*,*x_j_*) is a special function called the kernel. The coefficients *a_i_* and a_i_^*^ are the solutions of the quadratic problem:

(4) $$\begin{array}{l} \text{w}(a,{\text{a}}^{*}\text{)}=-ɛ\sum_{i=1}^{n}(a_{i}^{*}+a_{i})+\sum_{i=1}^{n}\left(a_{i}^{*}-a_{i})y_{i}-\frac{1}{2}\sum_{i,j=1}^{n}\left(a_{i}^{*}-a_{i}\right)(a_{j}^{*}-a_{j})k(x_{i},x_{j}\right)\\ \;\;\;\;\;\;\;\;\;\;\;\;\;\;\;\;\;\;\;\;\;\;\;\;\;\;\;\;\;\;\;\;\;\;\;\;\;\;\;\;\;\;\;\;0\leq a_{i},a_{i}^{*}\leq C,i=1,\cdots ,n,\\ \;\;\;\;\;\;\;\;\;\;\;\;\;\;\;\;\;\;\;\;\;\;\;\;\;\;\;\;\;\;\;\;\;\;\;\;\;\;\;\;\;\;\;\;\sum_{i=1}^{n}\left(a_{i}^{*}-a_{i}\right)=0 \end{array}$$

parameters C and ɛ can be chosen by the user. The “penalty parameter” C may be as high as infinity, while usual values for ɛ are 0.1 or 000.1.

The kernel function is used to convert the data into a higher-dimensional space in order to account for nonlinearities in the estimate function. A commonly used kernel is the Radial Basis Function (RBF) kernel:

(5) $$k(x,y)=\text{exp(}-\gamma {\left\|x-y\right\|}^{2}\text{)}$$

The parameter γ is selected by the user [46].

According to the program guide, two necessary steps had to be taken in advance: the scaling of input data and searching for best parameters. The input data (the descriptors selected by genetic-PLS) was compressed into [0.1, 0.9] through the formula:

(6) $$x*=\frac{x-x_{\text{min}}}{x_{\text{max}}-x_{\text{min}}}\times 0.8+0.1$$

where *x* was the original value, and x* is the scaled value. *x*_min_ and *x*_max_ are the corresponding minimum and maximum values of the descriptor variable, respectively.

There are three parameters to adjust the efficiency of Libsvm program: C, γ and ɛ. An autosearching program named “grid regression” was adopted. It could search for best parameters C, γ and ɛ through a leave-k-out cross validation method. Meanwhile, overfitting of training set could be prevented. Here a leave-25%-out cross validation was carried out. Manual searches were then performed around the leave-25%-out cross validation results to select the best parameters.

## 4. Results and Discussion

Six descriptors were selected from the initial 55 descriptors calculated by ADRIANA.Code after the genetic-PLS feature selection, which are N_rule5_, N_rot_, MW, LogS, TPSA and Acorr_Sigchg_3. They were used to build Model 1A and Model 1B by PLS and SVM, respectively.

Six descriptors were selected from the initial 52 descriptors calculated by Cerius^2^ after the genetic-PLS feature selection, which are N_rule5_, LogP, N_rot_, Jurs-FNSA-3, Jurs-RPCG and H_don_. They were used to build Model 2A and Model 2B by PLS and SVM, respectively.

Nine combined descriptors were taken from six selected ADRIANA.Code descriptors and six selected Cerius^2^ descriptors by a stepwise regression method. Nine combined descriptors were used to build Model 3A and Model 3B by PLS and SVM, respectively. The selected descriptors are shown in Table 1.

**Figure 5.**
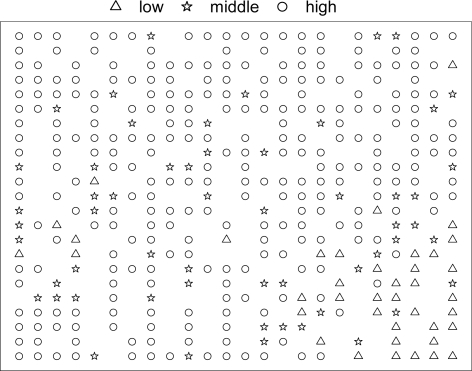
A rectangular KohNN map for 552 compounds obtained by 10 descriptors. ‘low’ means compounds with low Human intestinal absorption (HIA) in the range of [0 ∼ 29%], ‘middle’ means compounds with middle HIA in the range of [30 ∼ 79%], and ‘high’ means compounds with high HIA in the range of [80 ∼ 100%].

The pairwise correlation coefficients of the selected descriptors in each group have been estimated. None of the correlation coefficients is over 0.70. A rectangular KohNN with 24 × 23 was utilized with ten descriptors from six selected ADRIANA. Code descriptors and six selected Cerius^2^ descriptors as input vectors (two repeated descriptors were excluded before classification). The initial learning spans are 12 and 11.5, with an initial learning rate of 0.7 and a rate factor of 0.95. The initial weights are randomly initialized, and training was performed for a period of 1600 epochs in an unsupervised manner. A map was formed according to the ranges of Human intestinal absorption of the most frequently occupied neuron. The classification correctness rates were 89%. As indicated in Figure 5, compounds were mapped into Kohonen map according to their HIA ranges.

In the Kohonen map, 374 of a total of 552 neurons are occupied. Then, one object of each neuron was taken for the training set; for the conflict neurons, if the HIA values (%) of compounds in the same neuron had differences over 50, all compounds in this neuron were taken into training set; other objects were assigned as the test set. So 552 compounds were divided into a training set of 380 compounds and a test set of 172 compounds after the KohNN classification.

### 4.1. Partial Least Square (PLS) Models

Partial least square analysis was carried out with six selected ADRIANA.Code descriptors, six selected Cerius^2^ descriptors and nine combined descriptors to build Model 1A, Model 2A and Model 3A, respectively. 380 compounds in the training set were used to build models, 172 compounds in the test set were used to predict human intestinal absorption (HIA).

The equations were obtained as follows:

$$\text{HIA}\%=\sum (c_{i}D_{i})+D_{c}$$

In the equation, *D_i_* is a descriptor, and *c_i_* is its corresponding coefficient in the PLS model. *D_c_* is the constant in the equation. The corresponding coefficients are shown in Table 1.

For the training set of Model 1A, one component is abstracted, r =0.72, s=15.10, n=380 and *q*=0.70 and for the test set of Model 1A, *r* =0.83, *s*=13.06, *n*=172. (*r* is the correlation coefficient, s is the standard deviation), The root-mean-square (RMS) deviation of the calculated human intestinal absorption (%) of Model 1A is 18.79.

For the training set of Model 2A, one component is abstracted, *r* =0.73, *s*=14.67, *n*=380 and q=0.72 and for the test set of Model 2A, *r* =0.83, *s*=13.12, *n*=172. RMS of the calculated human intestinal absorption (%) of Model 2A is 18.67.

For the training set of Model 3A, one component is abstracted, *r* =0.74, *s*=14.97, *n*=380 and *q*=0.73 and for the test set of Model 3A, *r* =0.83, *s*=13.36, *n*=172. RMS of the calculated human intestinal absorption (%) of Model 3A is 18.18. The results are shown in Table 2.

### 4.2. Support Vector Machine (SVM) Models

Model 1B, Model 2B, and Model 3B were built by the Support Vector Machine with the Libsvm program [22]. Six selected ADRIANA. Code descriptors, six selected Cerius^2^ descriptors and nine combined descriptors were used to build Model 1B, Model 2B and Model 3B, respectively. For Model 1B, 380 compounds in the training set were used to train a Support Vector Machine (SVM) model, the option parameters were set as:*C* =32.0,*γ* =1.5,*ɛ* =0.125, and 172 compounds in the test set were used for prediction of HIA. For the training set, *r* =0.79, *s*=13.25, *n*=380, for the test set, *r* =0.87, *s*=10.98, *n*=172. RMS of the calculated HIA (%) of Model 1B is 16.68.

For Model 2B, 380 compounds in the training set were used to train a Support Vector Machine (SVM) model, the option parameters were set as:*C* =90.0,γ=1.0,*ɛ* =0.125 , and 172 compounds in the test set were used for prediction of HIA. For the training set, *r*=0.80, *s*=13.40, *n*=380, for the test set, *r* =0.89, *s*=9.72, *n*=172. RMS of the HIA (%) of Model 2B is 16.35.

For Model 3B, 380 compounds in the training set were used to train a Support Vector Machine (SVM) model, the option parameters were set as: *C* =32.0,γ =1.0,ɛ =0.125 , and 172 compounds in the test set were used for prediction of HIA. For the training set, *r*=0.81, *s*=12.50, *n*=380, for the test set, *r*=0.88, *s*=9.14, *n*=172. RMS of the HIA (%) of Model 3B is 16.00.

**Figure 6.**
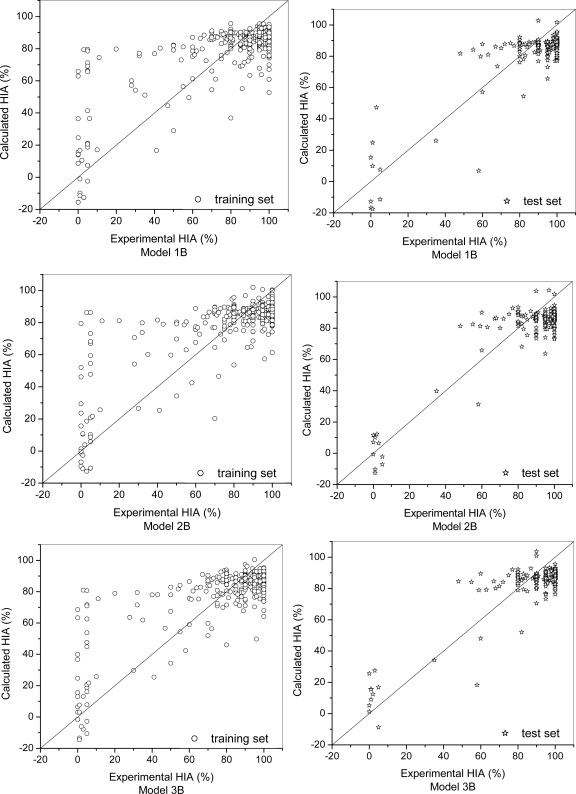
Calculated vs. Experimental values of human intestinal absorption (HIA) for the corresponding training sets and test sets of 552 compounds by Support Vector Machine (SVM) regression models. Model 1B are based on six selected ADRIANA.Code descriptors, Model2B are based on six selected Cerius^2^ descriptors and Model 3B are based on nine combined descriptors.

The results are shown in Table 2 and Figure 6.

According to the PLS and SVM prediction figures, all the models had a good prediction for high HIA (over 80%) compounds, but a poor prediction for low HIA (below 30%) ones. That was caused mainly by the unbalanced distribution of experimental HIA values. In the dataset, 71.7% of compounds had high HIA values over 80%; only 18.9% of compounds with HIA from 30% to 80%, and 9.4% compounds with HIA below 30%. Complicated mechanisms which are still unknown can lead to the irregular distribution in the low HIA area of the prediction figures, so the models were trained with biases to well-absorbed drugs. Great efforts are still needed to make to find more drugs with reliable and accurate experimental HIA during medium and low range. Hou’s model [23] with *r*=0.84 for training set of 455 compounds and *r*=0.90 for test of 98 compounds, which is still the best available. A descriptor named LogD in his model which is an extension of the LogP can response that. However, we have tried to explore to build proper prediction models of HIA with some the other descriptors (such as logS and 2D_Acorr_Sigchg_3) and some other methods (such as KonNN and SVM).

Models built by ADRIANA.CODE (Model 1A and Model 1B) and by Cerius^2^ (Model 2A and Model 2B) had similar performances: in the models of ADRIANA.CODE descriptors, best r=0.79 for training set, best r=0.87 for test set; in the models of Cerius^2^ descriptors, best r=0.80 for training set, best r=0.89 for test set. This indicates descriptors generated by each of them can provide enough information for HIA prediction. Some ADRIANA.CODE descriptors had showed their potentials for HIA prediction such as LogS and Acorr_Sigchg_3.

By comparison of the PLS models (Model 1A, Model 2A, Model 3A) and SVM models (Model 1B, Model 2B, Model 3B), it can be seen that SVM had obvious advantage in building HIA model. Taking test sets of three models as an example, r=0.83, r=0.83, r=0.83 in PLS models; r=0.87, r=0.89, r=0.88 in SVM models. It reveals the superiority of SVM as a non-linear method to linear methods. Genetic-PLS feature selection had been successfully applied to pick out the useful descriptors such as N_rule5_, TPSA, H_don_, LogP. But when dealing with some highly correlated descriptors, genetic-PLS can not recognize them. So high correlations should be eliminated before genetic-PLS selection.

## 5. Conclusions

The selected descriptors included some popular descriptors such as N_rule5_, TPSA, H_don_, LogP, and some unique ones such as Log*S* and Acorr_Sigchg_3. This indicated that σ charge values [33–34] which represent the influence of heteroatoms and the network of bonds in a computational scheme had a powerful ability in the prediction of HIA. Comparing the six models built with different descriptors and methods, it can be concluded that the models built with ADRIANA.CODE descriptors (Model 1A, Model 1B), Cerius^2^ descriptors (Model 2A, Model 2B), and the combination of them (Model 3A, Model 3B) had similar performances for the prediction of human intestinal absorption. Each of the descriptor generation software packages can work independently to build HIA prediction models.

The SVM method had shown a reliable ability in building effective models. This indicated that a non-linear method such as SVM is superior to a linear method such as PLS in building prediction models. The descriptors applied can be generated by calculation from the constitution of the molecules. For the 552 compounds, under the Windows XP (PM 790 MHZ) computer the descriptors used here can be calculated by ADRIANA.Code in 26 seconds. For the 552 compounds, under the Linux Redhat (IBMZ 2.5GHZ), all Cerius^2^ descriptors can be calculated in about 10 minutes. No experimental data such as additional descriptors are needed. Thus, the prediction models based on ADRIANA.Code descriptors can be used to work on larger datasets because of short computation time.

## Figures and Tables

**Table 1.** Selected descriptors and corresponding coefficients in the Partial Least Square models. Model 1A was based on six selected ADRIANA.Code descriptors, Model 2A was based on six selected Cerius^2^ and Model 3A was based on nine combined descriptors.

Model 1A		Model 2A		Model 3A	
descriptors	coefficient	descriptors	coefficient	descriptors	coefficient
N_rule5_	10.3161	N_rule5_	−10.0335	N_rule5_	8.4014
H_don_	2.8231	N_rot_	1.4978	N_rot_	1.2908
LogS	2.9385	LogP	1.4458	LogP	1.4358
MW	−0.0194	H_don_	2.7628	H_don_	2.5400
TPSA	0.1446	Jurs- FNSA3	85.0957	Jurs- FNSA3	97.3355
Acorr_Sigchg_3	14.5617	Jurs-RPCG	38.3653	Jurs-RPCG	28.8753
				LogS	1.7446
				MW	−0.0236
				Acorr_Sigchg_3	10.2598
*D_c_*	96.5824	*D_c_*	102.393	*D_c_*	105.466

Jurs- FNSA3 represents fractional charged partial surface areas [37].

Jurs-RPCG represents relative positive charge [37].

Acorr_Sigchg_3 is the third components of 2D autocorrelation coefficients for σ charge (where d=2)

**Table 2.** The prediction performances of 6 models: Partial Least Square (PLS) models and Support Vector Machine (SVM) models. Model 1A and Model 1B are based on six selected ADRIANA.Code descriptors; Model2A and Model2B are based on six selected Cerius^2^ descriptors; Model 3A and Model 3B are based on nine combined descriptors.

Model	Training set			Test set			RMS	
	n	r	s	n	r	s		
Model 1A	PLS	380	0.72	15.10	172	0.83	13.06	18.79
Model 1B	SVM	380	0.79	13.25	172	0.87	10.98	16.68
Model 2A	PLS	380	0.73	14.67	172	0.83	13.12	18.67
Model 2B	SVM	380	0.80	13.40	172	0.89	9.72	16.35
Model 3A	PLS	380	0.74	14.97	172	0.83	13.36	18.18
Model 3B	SVM	380	0.81	12.50	172	0.88	9.14	16.00
Hou’s model^17^		455	0.84	15.50	98	0.90	-	-

*n*: number of compounds;*r*: correlation coefficient; *s*: standard deviation.

RMS: root-mean-square (RMS) deviation for the whole model
